# Predictive Factors of Wound Healing and Limb Salvage After Successful Below-the-Knee Endovascular Angioplasty in Patients with Diabetic Foot Ulcer: A Retrospective Study

**DOI:** 10.3390/medicina61020277

**Published:** 2025-02-06

**Authors:** Chang Sik Shin, Kwon Cheol Yoo

**Affiliations:** 1 Department of Surgery, Uijeongbu Eulji University Hospital, Uijeongbu 11759, Republic of Korea; shincs22@gmail.com; 2 Department of Surgery, Chungbuk National University Hospital, Chungju 28644, Republic of Korea

**Keywords:** diabetic foot ulcer, wound healing, amputation

## Abstract

*Background and Objectives*: The primary objective of this study was to determine the predictive factors of limb salvage and wound healing in patients presenting with diabetic foot ulcers (DFUs) following successful below-the-knee endovascular angioplasty. *Materials and Methods*: Between January 2014 and January 2019, we retrospectively analyzed the wound healing and limb salvage rates of 85 patients (88 limbs) who underwent infra-popliteal endovascular treatment (EVT) for DFUs. Numerous variables were explored, including age, sex, comorbidities, and the scores from three DFU grading systems (Wagner grade, University of Texas (UT) grade and stage, and Wound, Ischemia, and foot Infection (WIfI) stage). Univariate and multivariate Cox proportional hazards analyses were conducted to determine the associations between adverse events and these variables. *Results*: During follow-up, 44 wounds healed completely, 47 limb amputations (major, 25; minor, 22) were required, and 17 limbs needed reintervention for wound healing. Nine patients who received treatment died of cardiovascular and cerebrovascular diseases, pneumonia, and other causes. At 1, 3, 6, 9, and 12 months, total wound healing rates were 4.6%, 16.9%, 27.5%, 34.5%, and 64.5%, respectively. At 6 months, 1 year, 2 years, and 5 years, amputation-free survival rates were 77.6%, 72.4%, 63.3%, and 63.3%, respectively. In multivariate Cox analyses, the UT grade and stage were associated with increased wound non-healing, while the UT grade and Wagner grade were associated with increased major lower-extremity amputation rates. Importantly, the UT grade was the only simultaneous risk factor predicting both wound healing and limb salvage. *Conclusions*: Despite successful below-the-knee angioplasty, a significant proportion of patients experienced wound non-healing and major amputation. The UT grade may serve as a predictor for both wound healing and limb salvage outcomes.

## 1. Introduction

According to a recent study, the prevalence of diabetes is 6.4% worldwide, and it is estimated that 10–15% of patients have a limb amputated every 20 s due to DFUs [1,2]. Globally, Type 2 DM is the most common form of diabetes, and its prevalence has risen dramatically over the past few decades. In Korea specifically, recent studies have shown that Type 2 DM increased from approximately 5% in 1988 to 15.3% by 2022, whereas Type 1 DM remains relatively rare (around 0.017–0.021%) [3,4]. As diabetic foot ulcers are more common in patients with Type 2 DM than Type 1 DM [2], understanding the predictors of limb salvage and wound healing has become increasingly important. EVT for DFU patients is widely performed because of its technical success rate of up to 90% and clinical success rates of around 70%, and in some cases, there are slightly higher rates of limb salvage and amputation-free survival compared to bypass surgery [5,6]. However, despite successful revascularization, many cases of failure to rescue the extremities have been reported. This suggests that the clinical success of bypass surgery or angioplasty is not solely dependent on the patency of the procedure, and that additional predictors—such as DFU classification (e.g., Wagner scale, UT grade and stage, WIfI stage) or pedal arch revascularization status (Type 1: both dorsalis pedis and plantar arteries patent; Type 2A: only dorsalis pedis artery patent; Type 2B: only plantar artery patent; Type 3: both dorsalis pedis and plantar arteries occluded)—can affect DFU outcomes [7,8,9,10]. The Rutherford classification, a representative indicator of ischemic wound evaluation, has traditionally been heavily used, but it relies strongly on measurements like ankle pressure and treadmill tests, which may insufficiently capture the complexity of diabetic foot wounds [11].

Hence, research on objective and comprehensive predictors of delayed wound healing and lower-extremity salvage in DFU patients is needed. Several indicators have been proposed [10,11,12,13] to predict delayed wound healing and lower-extremity salvage rates in these patients. Notably, the WIfI stage and UT grade/stage are objective systems that classify wound conditions into four stages according to ischemia and infection status, showing promise in predicting the delayed wound healing rate or limb salvage rate. However, only a small number of studies have evaluated how both of these factors influence wound healing and limb salvage in patients who undergo below-the-knee (BTK) angioplasty, leaving their full clinical utility uncertain.

## 2. Materials and Methods

### 2.1. Ethical Approval

This study was approved by the Institutional Review Board (IRB) of Eulji University Uijeongbu Hospital, Korea (IRB No.: UEMC 2022-05-006-001), and informed consent was not applicable because this is a retrospective study 

### 2.2. Definition

On duplex sonography, stenosis was defined when the peak systolic velocity (PSV) was twice or more that of a normal lesion. Ulcer wound healing was defined as complete re-epithelialization within 1 year. For lower-extremity amputation, amputation above the ankle was defined as major amputation, while amputation below the ankle was defined as minor amputation. Limb salvage was defined as the avoidance of major amputation. Successful EVT was defined as at least one patent runoff vessel.

#### 2.2.1. University of Texas (UT) Classification

The University of Texas (UT) classification system, introduced by Armstrong, Lavery, and Harkless in 1996, assesses ulcers based on depth, infection, and ischemia (four grades [0–3] × four stages [A–D]). (Table S1: Wound classification).

#### 2.2.2. Wagner Classification

The Wagner classification system, introduced by Wagner Jr. in 1981 [14], focuses on ulcer depth and the presence of osteomyelitis or gangrene (five grades [0–5]).

#### 2.2.3. WIfI Classification

The WIfI (Wound, Ischemia, and foot Infection) classification system, introduced by Mills et al. in 2014 [11], assesses ulcers based on wound, ischemia, and foot infection, providing a comprehensive view of the ulcer’s condition. (Table S2: WIFI amputation risk stage. Table S3: WIF amputation benefit stage).

### 2.3. Study Design and Population

From January 2014 to January 2019, the medical records of 85 patients (88 limbs) who underwent successful BTK EVT for Rutherford stages 5 and 6 DFUs at the Bundang Seoul University Hospital Department of Surgery were retrospectively reviewed. DFU was diagnosed by combining photographic evidence of the ulcer, preoperative studies (e.g., duplex sonography, ankle-brachial index [ABI], toe-brachial index [TBI]), blood test results, computed tomography of the lower extremities, and surgical findings such as angiographic results. Several risk factors known to affect diabetic foot outcomes (including DFU classification and pedal arch revascularization types 1, 2, and 3) were investigated. We excluded patients who had arterial stenosis or occlusion above the knee (femoral and iliac arteries), as well as those who underwent major amputation or bypass surgery at the same time. To minimize variability, we only enrolled patients who underwent successful EVT, the most frequently performed treatment in DFU patients, and excluded those who received bypass surgery.

### 2.4. Data Collection

We investigated DFU classification systems (Wagner grade, UT grade and stage, WIfI amputation risk stage, and WIfI intervention benefit stage). We also assessed factors such as ABI, toe pressure, transcutaneous oximetry, white blood cell (WBC) count, and C-reactive protein (CRP) to gauge the extent of ulcer infiltration, infection, and ischemic severity. The pedal arch was classified into three types according to revascularization status [10]. The revascularized vessel was further categorized as direct revascularization (DR) or indirect revascularization (IR) depending on whether the treated vessel directly supplied blood to the ulcer site [15,16,17].

### 2.5. Follow-Up

After the procedure, outpatient visits were conducted at intervals of 1, 3, and 6 months, then at 1 and 2 years. At each visit, ABI and TBI were measured, and ulcer status was confirmed through photographs.

### 2.6. Statistical Analysis

Age, sex, comorbidities, and ulcer characteristics were analyzed as potential risk factors for wound healing and major amputation (Table 1). Continuous variables are reported as mean ± SD and were compared using paired *t*-tests. Statistical significance was set at *p* < 0.05. Categorical variables were expressed as frequencies (%) and compared with the chi-squared test or Fisher’s exact test. Cox regression (univariate and multivariate) analyses were performed using SPSS Statistics 26 (IBM Corp, New York, NY, USA). The univariate Cox proportional hazards model was used to analyze variables (including DFU classification) in relation to major amputation and wound healing. All variables with *p* < 0.05 in the univariate analysis were included in the multivariate stepwise Cox analysis. Clinical outcomes, such as wound healing, limb salvage, reintervention-free survival, and mortality, were assessed by the Kaplan–Meier method. Subgroup analyses were performed using log-rank tests.

## 3. Results

No stenting was performed in this study population. Balloon angioplasty (plain balloon only) was performed in all patients, and aspirin or dual antiplatelet therapy was prescribed continuously post-procedure.

### 3.1. Clinical Characteristics

The clinical characteristics are summarized in Table 1. Patients were stratified according to wound healing status or lower-limb amputation. In total, 85 patients (88 limbs) were included. The mean age was 74.53 ± 17.81 years, and 62 patients (70%) were men. The mean body mass index (BMI) was 22.07 ± 0.43 kg/m^2^, the mean serum albumin level was 3.24 ± 0.60 g/dL, the mean glycated hemoglobin level was 7.25 ± 0.14%, the mean WBC count was 8493 ± 298/mL, the mean CRP was 3.03 ± 0.44 mg/dL, and the mean creatinine was 3.718 ± 0.14 mg/dL. Notable comorbidities included hypertension (70%), end-stage renal disease (ESRD) on dialysis (36.4%), coronary artery disease (47.7%), and cerebrovascular disease (21%). Aspirin was taken by 65% of patients, and insulin and cilostazol were each taken by 35%.

Ambulation status (33 [75%] vs. 21 [47.7%], *p* = 0.009) and ESRD on dialysis (11 [25%] vs. 21 [47.7%], *p* = 0.0027) differed significantly between the healing and non-healing groups. In the major amputation group, ESRD on dialysis was significantly more frequent (14 [56%] vs. 18 [28.6%], *p* = 0.01) compared with the limb salvage group. Although not statistically significant, CRP was higher in the major amputation group than in the limb salvage group (3.84 vs. 2.71 mg/dL, *p* = 0.061); conversely, the toe pressure was higher in the limb salvage group (33.45 mmHg) than in the major amputation group (19.25 mmHg) (*p* = 0.061). The difference in CRP may be related to a higher rate of infection in the major amputation group, while the higher ABI in that group could be attributable to advanced calcification in the lower-extremity arteries.

### 3.2. Ulcer Characteristics

Table 2 shows the ulcer characteristics. Of the 88 limbs, 44 wounds healed. The ulcer location was the toe (51 limbs, 58%), forefoot (29 limbs, 33%), heel (4 limbs, 4.5%), or leg/ankle (4 limbs, 4.5%). The ulcer type was an isolated ulcer in 35 limbs (40%), isolated gangrene in 22 limbs (25%), or a combination of ulcer and gangrene in 31 limbs (35%). A total of 61 limbs (70%) had DR, and 27 (30%) had IR. Pedal arch types were as follows: Type 1 (10 limbs, 11.4%), Type 2 (42 limbs, 47%), and Type 3 (36 limbs, 40%). According to the Wagner classification, 25 limbs (28.4%) were grade 1, 11 (12.5%) grade 2, 21 (23.9%) grade 3, 20 (22.7%) grade 4, and 11 (12.5%) grade 5. UT stages were A (14 limbs, 15.9%), B (7 limbs, 8%), C (33 limbs, 37.5%), and D (34 limbs, 38.6%). UT grades were 1 (35 limbs, 39.8%), 2 (45 limbs, 51.1%), and 3 (8 limbs, 9.1%). The WIfI amputation risk stages were stage 1 in 21 limbs (23.9%), stage 2 in 14 (15.9%), stage 3 in 25 (28.4%), and stage 4 in 28 (31.8%). The WIfI intervention benefit stages were stage 1 in 46 limbs (52.3%), stage 2 in 2 limbs (2.3%), stage 3 in 8 limbs (9.1%), and stage 4 in 32 limbs (36.4%). As shown in Table 2 limb salvage rates differed significantly by classification system. In the amputation group, most wounds presented with a high stage or high grade. Among the three DFU classifications, UT grade showed the most consistent association with both delayed wound healing and major amputation.

### 3.3. Risk Factor Analysis for Delayed Wound Healing and Limb Salvage Rate

Univariate and multivariate Cox proportional hazard analyses for wound healing and limb salvage are presented in Table 3. In the univariate Cox analysis, ambulatory status, preoperative toe pressure, UT grade and stage, Wagner grade, and the WIfI IB stage were significantly associated with wound healing. Stepwise multivariate Cox analysis identified the UT grade and stage as independent predictors of wound healing—meaning that the UT grade (wound depth) and stage (ischemic degree) critically affect wound healing.

In the major amputation group, non-ambulation, ESRD on dialysis, serum albumin level, toe pressure, BMI, Wagner grade, UT grade and stage, and the WIfI AR stage were significant risk factors. In stepwise multivariate Cox analysis, the UT grade and Wagner grade remained as independent predictors of major amputation. In multivariate analysis, the UT grade was the only factor that significantly predicted both wound healing and major amputation.

**Figure 1:**
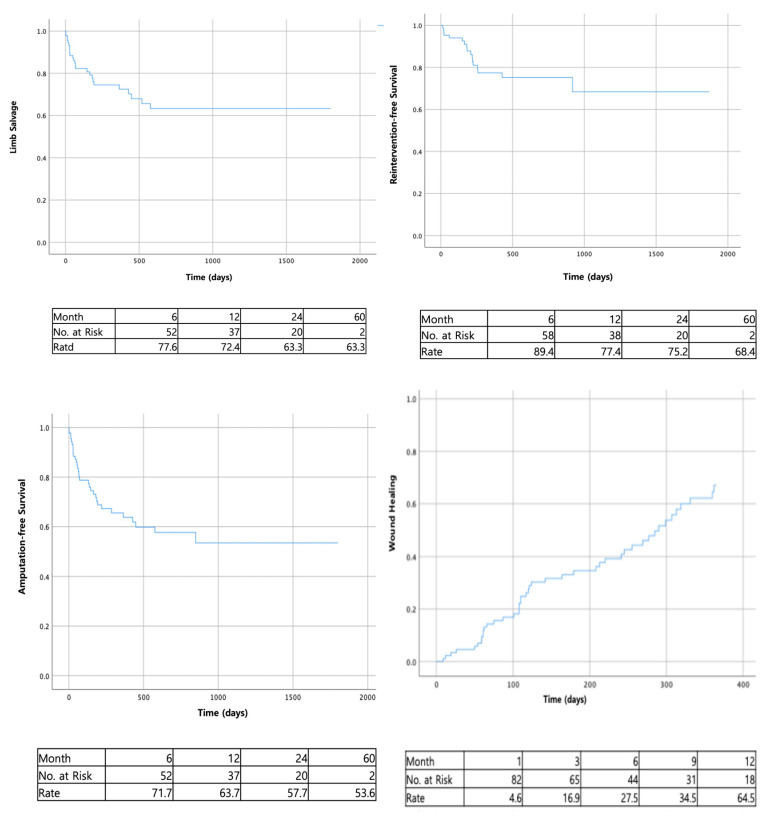
Kaplan–Meier life-table analysis of clinical outcomes after successful EVT.

**Figure 2:**
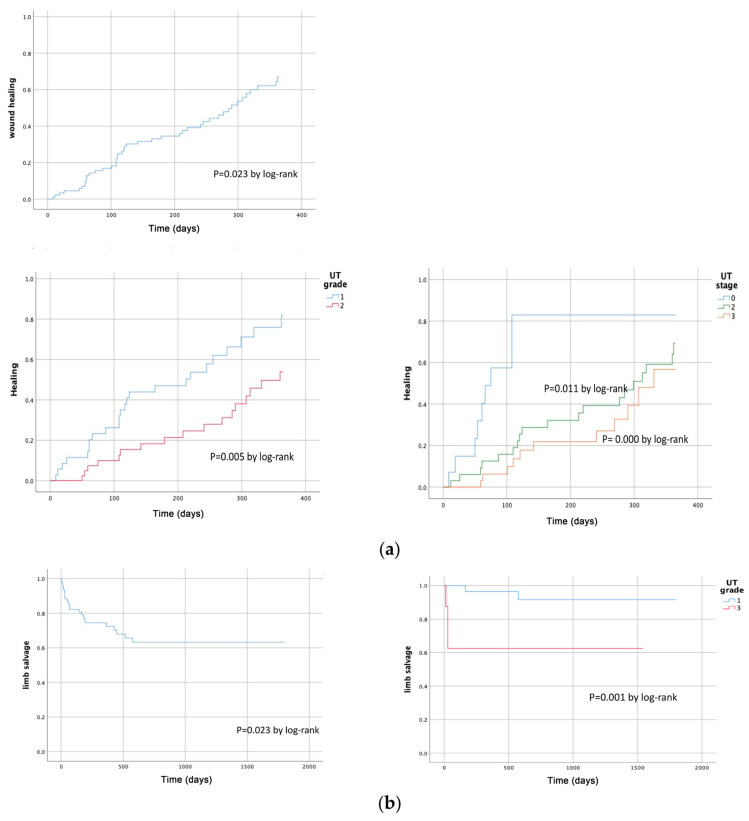
Kaplan–Meier life-table analysis of wound healing rates (**a**) and limb salvage rate (**b**) after EVT stratified by multiple variables(UT grade and stage, *p* < 0.05).

During follow-up, 44 wounds healed completely, 47 limbs underwent amputation (major, 25; minor, 22), and 17 limbs required reintervention for wound healing. Nine patients died from various causes (cardiovascular or cerebrovascular diseases, pneumonia, etc.). The reintervention-free survival rates at 6 months, 1 year, 2 years, and 5 years were 89.4%, 77.4%, 75.2%, and 68.4%, respectively. The amputation-free survival rates at 6 months, 1 year, 2 years, and 5 years were 71.7%, 63.7%, 57.7%, and 53.6%, respectively. The limb salvage rates were 77.6%, 72.4%, 63.3%, and 63.3%, respectively. Wound healing rates at 1, 3, 6, 9, and 12 months were 4.6%, 16.9%, 27.5%, 34.5%, and 64.5%, respectively (Figure 1). According to Kaplan–Meier survival analyses, risk factors affecting wound healing included UT grade 2 and UT stages B and C, while UT grades 2 and 3 affected major amputation (Figure 2; *p* < 0.05, log-rank test).

## 4. Discussion

In this study, the overall amputation rate was 43.4% in DFU patients after successful BTK percutaneous angioplasty, and the limb salvage rates at 6 months, 1 year, 2 years, and 5 years were 77.6%, 72.4%, 63.3%, and 63.3%, respectively. Compared with a previous study reporting that 2.7–30% of bypass-surgery patients required major amputation [18,19], or the study by Won et al. [20] (in which DFU patients had a 47% amputation rate with only 5% major amputations), our major amputation rate appears high. One reason may be that many patients in this cohort had advanced infection and ischemia before receiving proper management, possibly because they were initially evaluated by orthopedic or plastic surgery and were already being considered for amputation.

Previous literature has identified ESRD on dialysis and infectious wounds as important factors for limb salvage [6], but relatively few studies have focused on limb salvage predictors after BTK EVT in Korea [12,13]. A recent study found that hemodialysis is also an independent predictor of lower-limb salvage after BTK EVT, similar to bypass surgery [21]. Other risk factors for limb amputation include older age, male sex, stroke, ischemic heart disease with hypertension, nephropathy, longer diabetes duration, and sensory neuropathy [22,23]. In our current study, the DFU classification—particularly the UT grade—emerged as the only significant risk factor in both the amputation and wound healing groups.

In clinical practice, incomplete wound healing after EVT does not necessarily lead to major amputation. Thus, major amputation alone may not capture the entire scope of DFU outcomes. Wound healing can also be a key factor influencing patient quality of life. While some studies have analyzed limb amputation or wound healing separately, few have examined both outcomes simultaneously in DFU patients undergoing EVT. In our study, we employed the University of Texas (UT), Wagner, and WIFI wound classification systems to evaluate diabetic foot ulcers. When compared, all three systems showed positive trends with increased amputation rates. However, the UT system, which includes stages, proved to be a superior predictor of outcomes. A study published in Diabetes Care (2001) supports this finding, concluding that increasing stages in the UT system, regardless of grade, are associated with a higher risk of amputation and prolonged healing time [14]. The Wagner classification system primarily categorizes wounds based on depth, with each stage providing specific criteria regarding the appearance and condition of the wound. Conversely, the University of Texas (UT) classification system includes infection and necrosis status, offering a more comprehensive reflection of the complexity and urgency of treatment. While the Wagner system intuitively divides the severity of wounds, the UT system captures critical details necessary for treatment, including infection and necrosis status. Both systems are essential for treating diabetic foot ulcers, but the UT system can be considered more detailed and practical in guiding treatment decisions. This paper explains that the two wound classification systems evaluate diabetic foot ulcers based on different criteria and play a crucial role in therapeutic approaches. The systems are complementary and should be selected according to the wound characteristics and treatment needs. ‘Penetrating to tendon or capsule (UT grade II) and to bone or joint (UT grade III) present a complex set of issues—including compromised vascular supply, elevated risk of infection, and a chronic inflammatory environment—which collectively delay wound healing and increase the risk of amputation’. As wounds become deeper, blood flow is restricted, leading to reduced tissue oxygenation, and the risk of infection (e.g., osteomyelitis) rises when bone or joint structures are involved. In addition, chronic inflammation impairs tissue regeneration and complicates mechanical offloading, further delaying wound improvement. Therefore, procedures such as revascularization, infection control, debridement of necrotic tissue, offloading, and advanced regenerative therapies must be integrated, ideally through a multidisciplinary approach to optimize outcomes. Consequently, higher UT grades indicate greater wound depth and more complex complications, making early detection and aggressive intervention critical for improving wound healing rates and reducing the risk of amputation. Indeed, if both the wound depth and ischemic status are assessed via the UT grade and stage, more accurate predictions of wound healing and major amputation may be achieved.

This study has several limitations. First, it was retrospective and included a relatively small number of patients, because (1) many patients were excluded to focus on a single treatment method, and (2) long-term treatment is challenging, leading to loss to follow-up. Second, our study is a retrospective analysis based on chart reviews and treatment imaging results. Due to the limitations inherent in chart reviews, we were unable to accurately categorize the type and duration of diabetes mellitus (DM). Specifically, data on HbA1c levels, the duration of diabetes, and the type of diabetes were not consistently available in the charts reviewed. This limitation makes it challenging to provide a detailed breakdown. Additionally, the exact date of the first diagnosis of diabetes is not specified, and HbA1c measurements, which could have served as a reference point, were not routinely conducted prior to treatment. As a result, it is difficult to accurately reflect these metrics in our statistics. Consequently, we are unable to provide precise figures for the numbers of Type 1 (T1D) and Type 2 (T2D) diabetes cases or their respective durations in the tables. Additionally, as a retrospective chart review study analyzing surgical treatment and visual wound assessments, many charts were missing information regarding medications or antibiotics used during treatment, which prevented us from including these data in the study. Additionally, in the case of diabetic foot ulcers, the characteristics can vary greatly among patients, making it challenging to establish uniform classification criteria. Thus, the first priority should be to create clear and standardized criteria for classification. Additionally, further research should include a detailed investigation of the type and duration of diabetes, the medications being taken, associated complications, and blood test results reflecting the severity of diabetes. This would provide a more comprehensive understanding of the factors that exacerbate diabetic foot ulcers.

Finally, by extending the study duration, we could recruit a larger patient cohort and expand the timeframe over which treatment outcomes are assessed. This would allow for more robust data collection and analysis, ultimately leading to more effective treatment protocols and better management strategies for patients with diabetic foot ulcers.

## 5. Conclusions

The UT grade, which objectively reflects the severity of diabetic foot ulcers, can predict wound healing and limb salvage in patients undergoing BTK EVT. Despite successful below-the-knee angioplasty, a significant proportion of patients still experience non-healing wounds and major amputation. Identifying high-risk patients using the UT classification could guide more timely and targeted interventions, potentially improving long-term outcomes.

## Figures and Tables

**Table 1:** Patients’ demographics per group (healed vs. non-healed, (**a**), and amputation vs. non-amputation, (**b**)).

(**a**)				
**Variables**	**Healed (*n* = 44)**	**Non-Healed (*n* = 44)**	**Total (*n* = 88)**	***p*** **-Value**
Age (years)	74.659	74.409	74.53 ± 1.94	0.215
Male, *n* (%)	34 (77.3%)	28 (63.6%)	62 (70%)	0.161
BMI	22.73	21.41	22.07 ± 0.43	0.129
HbA1c	7.17	7.33	7.25 ± 0.18	0.070
Hb	10.81	10.32	10.56 ± 0.18	0.907
WBC	8472	8515	8493 ± 298	0.756
CRP	3.08	2.99	3.03 ± 0.46	0.805
Creatine	2.55	4.78	3.67 ± 0.71	0.100
Albumin	3.39	3.10	3.24 ± 0.06	0.239
ABI				0.645
>0.8	25 (62.5%)	21 (58.3%)	46 (60.5%)	
0.6~0.79	6 (15%)	5 (13.9%)	11 (14.5%)	
0.4~0.59	7 (17.5%)	5 (13.9%)	12 (15.8%)	
<0.4	7 (9.2%)	5 (13.9%)	7 (9.2%)	
TP				0.324
>60	6 (15.8%)	3 (7.5%)	9 (11.5%)	
40~59	10 (26.3%)	7 (17.5%)	17 (21.8%)	
30~39	6 (15.8%)	5 (12.5%)	11 (14.1%)	
<30	16 (42.1%)	25 (32.5%)	41 (52.6%)	
TCPO_2_				0.511
>40	11 (28.2%)	12 (30%)	23 (29.1%)	
30~39	7 (17.9%)	11 (27.5%)	18 (22.8%)	
<30	21 (53.8%)	17 (42.5%)	38 (48.1%)	
HTN	35 (79.5%)	35 (79.5%)	70 (79.5%)	1.0
CAD	19 (43.2%)	23 (52.3%)	42 (47.7%)	0.522
CVD	8 (18.2%)	11 (25%)	19 (21.6%)	0.605
**ESRD on dialysis**	**11 (25%)**	**21 (47.7%)**	**32 (36.4%)**	**0.027**
Dyslipidemia	10 (22.7%)	8 (18.2%)	18 (20.5%)	0.597
Smoking	14 (31.8%)	12 (27.3%)	26 (29.5%)	0.640
**Ambulation**	**33 (75%)**	**21 (47.7%)**	**54 (61.4%)**	**0.009**
Aspirin	26 (59.1%)	32 (72.7%)	58 (65.9%)	0.177
Cilostazol	14 (31.8%)	17 (38.6%)	31 (35.2%)	0.503
(**b**)				
**Variables**	**Amputation (*n* = 25)**	**Non-Amputation** **(*n* = 63)**	**Total (*n* = 88)**	***p*** **-Value**
Age (years)	73.8	74.82	74.53 ± 1.17	0.549
Male, *n* (%)	17 (68%)	45 (71.4%)	62 (70%)	0.751
BMI	20.34	22.75	22 ± 0.43	0.155
HbA1c	7.14	7.30	7.25 ± 0.14	0.661
Hb	10.11	10.75	10.56 ± 0.18	0.134
WBC	8792	8374	8493 ± 298	0.608
CRP	3.88	2.69	3.03 ± 0.44	0.061
Creatine	3.97	3.54	3.68 ± 0.71	0.407
Albumin	3.00	3.34	3.24 ± 0.06	0.174
ABI				0.621
>0.8	16 (69.6%)	30 (56.6%)	46 (60.5%)	
0.6~0.79	2 (8.7%)	9 (17%)	11 (14.5%)	
0.4~0.59	4 (17.4%)	8 (15.1%)	12 (15.8%)	
<0.4	1 (4.3%)	6 (11.3%)	7 (9.2%)	
TP				0.80
>60	1 (4.2%)	8 (14.8%)	9 (11.5%)	
40~59	2 (8.3%)	15 (27.8%)	17 (21.8%)	
30~39	4 (16.7%)	7 (13%)	11 (14.1%)	
<30	17 (70.8%)	24 (44.4%)	41 (52.6%)	
TCPO_2_				0.498
>40	7 (30.4%)	16 (28.6%)	23 (29.1%)	
30~39	7 (30.4%)	11 (30.4%)	18 (22.8%)	
<30	9 (39.1%)	29 (51.8%)	38 (48.1%)	
HTN	18 (72%)	52 (82.5%)	70 (79.5%)	0.269
CAD	12 (48%)	30 (47.6%)	42 (47.7%)	0.974
CVD	8 (32%)	11 (17.5%)	19 (21.6%)	0.135
**ESRD on dialysis**	**14 (56%)**	**18 (28.6%)**	**32 (36.4%)**	**0.016**
Dyslipidemia	4 (16%)	14 (22.2%)	18 (20.5%)	0.514
Smoking	9 (36%)	17 (27%)	26 (29.5%)	0.403
Ambulation	12 (48%)	42 (66.7%)	54 (61.4%)	0.105
Insulin	10 (40%)	21 (33.3%)	31 (35.2%)	0.555
Aspirin	17 (68%)	41 (65.1%)	58 (65.9%)	0.794
Cilostazol	11 (44%)	20 (31.7%)	31 (35.2%)	0.278

(BMI: Body Mass Index, WBC: white blood cell, CRP: C-Reactive Protein, ABI: Ankle brachial Index, TP: Toe Pressure, TCPO_2_: Transcutaneous Oxygen Pressure, HTN: Hypertension, CAD: Coronary Artery Disease, CVD: CerebroVascular disease, ESRD: End Stage of Renal Disease).

**Table 2:** Baseline lesion characteristics before angioplasty per group (healed vs. non-healed, (**a**), and amputation vs. non-amputation, (**b**)).

(**a**)				
**Variables**	**Healed (*n* = 44)**	**Non-Healed (*n* = 44)**	**Total (*n* = 88)**	***p*** **-Value**
Wound location				0.309
Toe	27 (61.4%)	24 (54.5%)	51 (58%)	
Dorsal or plantar	15 (34.1%)	14 (31.8%)	29 (33%)	
Ankle	2 (4.5%)	2 (4.5%)	4 (4.5%)	
Heel	0	4 (9.1%)	4 (4.5%)	
Wound type				0.146
Ulcer	22 (50%)	13 (29.5%)	35 (39.8%)	
Gangrene	9 (20.5%)	13 (29.5%)	22 (25%)	
Combined	13 (29.5%)	18 (40.9%)	31 (35.2%)	
Vascularization				0.106
DR	34 (77.3%)	27 (61.4%)	61 (69.3%)	
IR	10 (22.7%)	17 (38.6%)	27 (30.7%)	
Pedal arch classification				0.360
Classification 1	7 (15.9%)	3 (6.8%)	10 (11.4%)	
Classification 2	21 (47.7%)	21 (47.7%)	42 (47.7%)	
Classification 3	16 (36.4%)	20 (45.5%)	36 (40.9%)	
Wagner				0.084
Wagner 1	17 (38.6%)	8 (18.2%)	25 (28.4%)	
Wagner 2	6 (13.6%)	5 (11.4%)	11 (12.5%)	
Wagner 3	9 (20.5%)	12 (27.3%)	21 (23.9%)	
Wagner 4	10 (22.7%)	10 (22.7%)	20 (22.7%)	
Wagner 5	2 (4.5%)	9 (20.5%)	11 (12.5%)	
**UT grade**				**0.017**
**Grade 1**	**24 (54.5%)**	**11 (25%)**	**35 (39.85%)**	
**Grade 2**	**17 (38.6%)**	**28 (63.6%)**	**45 (51.1%)**	
**Grade 3**	**3 (6.85%)**	**5 (11.4%)**	**8 (9.1%)**	
UT stage				0.055
Stage A	10 (22.7%)	4 (9.1%)	14 (15.9%)	
Stage B	4 (9.1%)	3 (6.8%)	7 (8%)	
Stage C	19 (43.2%)	14 (31.8%)	33 (37.5%)	
Stage D	11 (25%)	23 (52.3%)	34 (38.6%)	
WIFI AR				0.101
Stage 1	14 (31.8%)	7 (15.9%)	21 (23.9%)	
Stage 2	8 (15.9%)	6 (13.6%)	14 (15.9%)	
Stage 3	13 (29.5%)	12 (27.3%)	25 (28.4%)	
Stage 4	9 (20.5%)	19 (43.2%)	28 (31.8%)	
WIFI IB				0.356
Stage 1	25 (56.8%)	21 (47.7%)	46 (52.3%)	
Stage 2	2 (4.5%)	0	2 (4.5%)	
Stage 3	4 (9.1%)	4 (9.1%)	8 (9.1%)	
Stage 4	13 (29.5%)	19 (43.2%)	32 (36.4%)	
(**b**)				
**Variables**	**Major Amputation** **(*n* = 25)**	**Non-Amputation** **(*n* = 63)**	**Total (*n* = 88)**	***p*** **-Value**
Wound location				0.788
Toe	16 (64%)	35 (55.6%)	51 (58%)	
Dorsal or plantar	8 (32%)	21 (33.3%)	29 (33%)	
Ankle	1 (4%)	3 (4.8%)	4 (4.5%)	
Heel	0	4 (6.3%)	4 (4.5%)	
Wound type				0.140
Ulcer	6 (24%)	29 (46%)	35 (39.8%)	
Gangrene	7 (28%)	15 (23.8%)	22 (25%)	
Combined	12 (48%)	19 (30.2%)	31 (35.2%)	
Vascularization				0.088
DR	14 (56%)	47 (74.6%)	61 (69.3%)	
IR	11 (44%)	16 (25.4%)	27 (30.7%)	
Pedal arch classification				0.907
Classification 1	3 (12%)	7 (11.1%)	10 (11.4%)	
Classification 2	11 (44%)	31 (49.2%)	42 (47.7%)	
Classification 3	11 (44%)	25 (39.7%)	36 (40.9%)	
**Wagner**				**0.010**
**Wagner 1**	**2 (8%)**	**23 (36.5%)**	**25 (28.4%)**	
**Wagner 2**	**3 (12%)**	**8 (12.7%)**	**11 (12.5%)**	
**Wagner 3**	**5 (20%)**	**16 (25.4%)**	**21 (23.9%)**	
**Wagner 4**	**11 (44%)**	**9 (14.3%)**	**20 (22.7%)**	
**Wagner 5**	**4 (16%)**	**7 (11.1%)**	**11 (12.5%**	
**UT grade**				**0.001**
**Grade 1**	**2 (8%)**	**33 (52.4%)**	**35 (39.8%)**	
**Grade 2**	**20 (80%)**	**25 (39.7%)**	**45 (51.1%)**	
**Grade 3**	**3 (12%)**	**5 (7.9%)**	**8 (9.1%)**	
**UT stage**				**0.004**
**Stage A**	**1 (4%)**	**13 (20.6%)**	**14 (15.9%)**	
**Stage B**	**1 (4%)**	**6 (9.5%)**	**7 (8%)**	
**Stage C**	**6 (24%)**	**27 (42.9%)**	**33 (37.5%)**	
**Stage D**	**17 (68%)**	**17 (27%)**	**34 (38.6%)**	
**WIFI AR**				**0.025**
**Stage 1**	**1 (4%)**	**20 (31.7%)**	**21 (23.9%)**	
**Stage 2**	**3 (12%)**	**11 (17.5%)**	**14 (15.9%)**	
**Stage 3**	**10 (40%)**	**15 (23.8%)**	**25 (28.4%)**	
**Stage 4**	**11 (44%)**	**17 (27%)**	**28 (31.8%)**	
WIFI IB				0.683
Stage 1	15 (60%)	31 (49.2%)	46 (52.3%)	
Stage 2	0	2 (3.2%)	2 ( (2.3%)	
Stage 3	1 (4%)	7 (11.1%)	8 (9.1%)	
Stage 4	9 (36%)	23 (36.5%)	32 (36.4%)	

**Table 3:** Predictors of major amputation and wound healing in the univariate and multivariate Cox proportional hazard analysis.

Variable	Wound Healing			Amputation		
		Multivariate Analysis		Univariate Analysis	Multivariate Analysis	
	*p*-Value	Hazard Ratio	*p*-Value	*p*-Value	Hazard Ratio	*p*-Value
Age	0.921			0.349		
Male	0.264			0.633		
HTN	0.727			0.343		
DM	0.941			0.777		
CVD	0.767			0.075		
CAD	0.786			0.797		
Dyslipidemia	0.133			0.586		
ESRD on dialysis	0.053		0.264	**0.007**		0.220
Ambulation	**0.017**		0.270	**0.032**		0.436
Smoking	0.540			0.498		
Cilostazol	0.724			0.438		
Albumin	0.091			**0.018**		0.256
CRP	0.459			0.060		0.908
ABI	0.858			0.130		
Toe pressure	**0.031**		0.471	**0.040**		0.438
TCPO_2_	0.065			0.918		
Hb	0.847			0.546		0.098
BMI	0.206			**0.006**		0.158
Wound location	0.721			0.749		
Wagner	**0.047**		0.410	**0.003**		0.070
**UT grade (ref.1)**	**0.016**		**0.004**	**0.000**		**0.007**
**2**		**0.192 (0.069~0.534)**	**0.002**		**25.72 (3.41~193.88)**	**0.002**
**3**		0.442 (0.085~2.297)	0.332		**23.81 (2.34~232.50)**	**0.006**
**UT stage (ref. A)**	**0.003**		**0.003**	**0.000**		0.464
**B**		**0.141 (0.028~0.698)**	**0.016**			
**C**		**0.179 (0.069~0.464)**	**0.000**			
**D**		0.422 (0.139~1.283)	0.128			
WIFI AR stage	0.149			**0.023**		0.282
WIFI IB stage	**0.050**		0.694	0.567		
Vascularization	0.342			0.129		
Pedal arch class	0.145			0.986		

## Data Availability

Data available in a publicly accessible repository. The original data presented in the study are openly available in this article.

## Supplementary Materials

The following supporting information can be downloaded at: https://www.mdpi.com/article/10.3390/medicina61020277/s1, Table S1: Wound classification; Table S2: WIFI amputation risk stage; Table S3: WIF amputation benefit stage.
