# Supplementary material for: Predictive Factors of Wound Healing and Limb Salvage After Successful Below-the-Knee Endovascular Angioplasty in Patients with Diabetic Foot Ulcer: A Retrospective Study
Source: Medicina (Kaunas). 2025 Feb 6;61(2):277. doi: 10.3390/medicina61020277 (PMC11857322; doi:10.3390/medicina61020277)
Supplement: Supplementary file 1 [file medicina-61-00277-s001.zip › medicina-3413877-supplementary.pdf]

medicina

MDPI

Supplementary Table S1. Wound classification

Wagner 0	pre- or post-ulcerative lesion
Wagner 1	partial/full-thickness ulcer
Wagner 2	probing to tendon or capsule
Wagner 3	deep with osteitis
Wagner 4	partial foot gangrene
Wagner 5	whole foot gangrene
UT grade 1	superficial wound not involving tendon, capsule, or bone
UT grade 2	wound penetrating tendon or capsule
UT grade 3	wound penetrating bone or joint
UT stage A	clean wounds
UT stage B	non-ischaemic infected wounds
UT stage C	ischaemic non-infected wounds
UT stage D	ischaemic infected wounds
WIFI	
Wound 1	minor tissue loss. Salvageable with simple digital amputation (1 or 2 digits) or skin coverage with no gangrene

Wouud 2	major tissue loss salvageable with multiple (>3) digital amputations or standard Transmetatarsal amputation skin coverage. And gangrenous changes limited to digits
Wound 3	extensive tissue loss salvageable only with a complex foot reconstruction or nontraditional TMA (Chopart or Lisfranc); flap coverage or complex wound management needed for large soft tissue defect. And extensive gangrene involving forefoot
Ischemia 0	ABI ≥ 0.8 or TP, TcPO2 ≥ 60mm Hg or ankle systolic pressure> 100mm Hg)
Ischemia 1	ABI : 0.6-0.79 or TP, TcPO2 40-59mm Hg, or ankle systolic pressure: 70~100mm Hg
Ischemia 2	ABI : 0.4-0.59 or TP, TcPO 30-39mm Hg, or or ankle systolic pressure :50~70mm Hg
Ischemia 3	ABI ≤ 0.39 or TP, TcPO2 < 30mm Hg, or or ankle systolic pressure < 50mm Hg)
Infection 0	no infection
Infection 1	infection present, as defined by the presence of at least 2 of the following items: Local swelling or induration, Erythema >0.5 to <2 cm around the ulcer, Local tenderness or pain, Local warmth, Purulent discharge (thick, opaque to white, or sanguineous secretion
Infection 2	Local infection (as described above) with erythema >2 cm, or involving 2 structures deeper than skin and subcutaneous tissues (eg, abscess,

	osteomyelitis, septic arthritis, fasciitis), and no systemic inflammatory response signs
Infection 3	Local infection with the signs of SIRS, as manifested by two or more of the following: Temperature >38°C or <36°C, Heart rate >90 beats/min, Respiratory rate >20 breaths/min or PaCO2 <32 mm Hg, White blood cell count >12,000 or <4000 cu/mm or 10% immature

Supplementary Table S2. WIFI amputation risk stage

	Ischemia - 0				Ischemia -1					Ischemia-3				Ischemia-4			
W0	1	1	2	3	4	2	3	4		2	2	3	4	2	3	3	4
W1	1	1	2	3	4	2	3	4		2	3	4	4	3	3	4	4
W2	2	2	3	4	3	3	4	4		3	4	4	4	4	4	4	4
W3	3	3	4	4	4	4	4	4		4	4	4	4	4	4	4	4

Supplementary Table S3. WIF amputation benefit stage

	Ischemia - 0				Ischemia -1					Ischemia-3				Ischemia-4			
W0	1	1	1	1	1	2	2	3		2	2	3	3	3	4	4	4
W1	1	1	1	1	2	3	3	3		3	4	4	4	4	4	4	4
W2	1	1	1	1	3	3	4	4		4	4	4	4	4	4	4	4
W3	1	1	1	1	3	3	3	4		4	4	4	4	4	4	4	4
